# Differences in the Malariometric Indices of Asymptomatic Carriers in Three Communities in Ibadan, Nigeria

**DOI:** 10.1155/2014/509236

**Published:** 2014-12-23

**Authors:** Olukemi K. Amodu, Adesola O. Olumide, Obioma C. Uchendu, Folakemi A. Amodu, Olayemi O. Omotade

**Affiliations:** ^1^Institute of Child Health, College of Medicine, University of Ibadan, Ibadan 200001, Nigeria; ^2^Department of Community Medicine, College of Medicine, University of Ibadan, Ibadan 200001, Nigeria

## Abstract

This study was conducted to determine the malariometric indices of children in three different settings in Ibadan, Nigeria. Children were recruited from an urban slum (Oloomi) and a periurban (Sasa) and a rural community (Igbanda) in Ibadan. Children aged between 2 and 10 years were randomly selected from primary schools in the urban and periurban areas. In the rural community, children were recruited from the centre of the village. A total of 670 (55.0%) out of 1218 children recruited were positive for malaria parasitaemia. The urban population had the highest proportion of children with malaria parasitaemia. Splenomegaly was present in 31.5%, hepatomegaly in 41.5%, hepatosplenomegaly in 27.5%, and anaemia in 25.2% of the children. The parasite density was not significantly different among children in the three communities. Children in the rural community had the highest mean PCV of 34.2% and the lowest rates of splenomegaly (6.1%), hepatomegaly (7.6%), and hepatosplenomegaly (4.6%). The spleen rates, liver rates, and presence of hepatosplenomegaly and anaemia were similar in the urban and periurban communities. The malariometric indices among the asymptomatic carriers were high, especially in the urban slum. This stresses the need for intensified efforts at controlling the disease in the study area.

## 1. Introduction

Malaria remains a leading cause of illness and death in Sub-Saharan Africa with the greatest risk seen in children under the age of five and pregnant women. About 50% of Nigerians are estimated to have at least one episode of the disease each year with over 200,000 deaths in children annually [1].

Malaria is a poverty-related disease and thus is mostly associated with the rural areas in Africa. Many studies have shown that malaria transmission is generally lower in urban compared with rural areas [2, 3]. This has been largely attributed to such factors as higher human density, better quality housing, and better access to healthcare facilities, and consequently they suffer lower malaria morbidity and mortality in urban areas [2]. However, in many urban populations, malaria remains a leading cause of morbidity and mortality [4–6].

Malaria transmission is influenced by many factors including the environment, the vector, and consequently the human host. Malaria risk is often unevenly distributed across different communities, with variations in malariometric indices between and within many areas [7–9]. In areas of intense transmission, clinical presentations of malaria in the population include a low-grade infection, asymptomatic parasitaemia (AsyM). These asymptomatic carriers constitute a transmission reservoir of gametocyte and thus influence transmission and the burden of malaria [10, 11]. Estimates of malaria burden are based on malariometric indices like prevalence of malaria parasitaemia, spleen rate, and anaemia in defined risk groups, particularly children of school age. An understanding of the malaria burden in a given setting is important for health planning, policy development, and control interventions. This study was carried out to determine and compare the malaria burden in three communities (urban, periurban, and rural) in Ibadan.

## 2. Subjects and Methods

Cross-sectional studies were carried out in Ibadan, a city in southwest of Nigeria, a holoendemic area for malaria. Children were recruited from three communities, Oloomi, an urban slum (with crowded housing and poor environmental sanitation) in the central area of Ibadan, Sasa (a periurban community with features of an urban slum but with better spaced housing and rural characteristics), and Igbanda (a rural community within Ojoo area in Ibadan). Children aged 2–10 years attending three primary schools in the urban area and one primary school in the periurban area were considered eligible and were recruited into the study. In the rural community, children were recruited from the centre of the village. They were selected using a multistage stratified cluster sampling technique. A total number of 1182 children were recruited.

The study was carried out between May 2007 and September 2009. Ethical approval was obtained from the joint University of Ibadan-University College Hospital, Ibadan Ethical Committee, and from the Oyo State Health Board. Informed consent was obtained from the parents or guardian of the children prior to recruitment. Demographic information and clinical history were obtained from care-givers of the children. Clinical examination—general physical examination including anthropometric measurements (weight and height), measurement of temperature, and examination for pallor and jaundice—was done for all the children. In addition, they were assessed for palpable enlarged spleen and liver and categorized as “no enlargement,” “presence of hepatomegaly only,” “presence of splenomegaly only,” and “presence of hepatosplenomegaly” following standard methods [12–15]. Children were excluded from the study if informed consent was not obtained from a relative. All children with uncomplicated malaria were treated according to the National Malaria Treatment Policy [16]. Thick blood smears stained with Giemsa were prepared for each child and examined for trophozoites of* P. falciparum*. Parasite densities were calculated based on assumed total WBC of 8000/uL. Blood films were defined as negative if there were no asexual forms of* P. falciparum* in 100 high power fields of a thick film. Blood was also sampled for haematocrit or packed cell volume (PCV).

Statistical analysis was done with the SPSS 16.0 for Windows (SPSS Inc., Chicago, USA). The children were grouped as “younger children (≤60 months)” and “older children (>60 months)” for analysis. The prevalence of malaria parasitaemia, spleen rate, liver rates, and anaemia (PCV < 30%) was calculated as a proportion of children with those indices. Chi-square was used for association between categorical variables. Statistical significance was set at *P* < 0.05.

## 3. Results

A total of 1182 children were recruited. The rural community had the lowest number of males (*P* = 0.033) and the lowest proportion of younger children, *P* < 0.0001 (Table 1). Overall, 657 (55.6%) were positive for malaria parasitaemia (mp) without symptoms or history of illness within the last two weeks and these were classified as asymptomatic (AsyM). The median age of the AsyM population was 46.0 months; 51.8% were males and 55.6% were younger children. The urban population had the highest proportion of children with malaria parasitaemia 60.4% (411/681) compared with the periurban population with 53.6% (119/222) and the rural population with 45.5% (127/279); *P* = 0.000. The haematocrit/packed cell volume (PCV) of 11 of them (1.6%) could not be obtained due to breakage of capillary tubes. These were excluded from analysis related to anaemia and mean haematocrit estimation. AsyM population were assessed for malariometric indices such as parasite density, haematocrit, splenomegaly (spleen enlargement), hepatomegaly (liver enlargement), and hepatosplenomegaly (both liver and spleen enlargement). Splenomegaly was present in 31.5% of the population, hepatomegaly in 41.6% of the population, and hepatosplenomegaly in 27.4% of the population. The mean parasite density for the total population was 676/*μ*L and the mean haematocrit/packed cell volume (PCV) was 32.5 ± 5.7%. Anaemia (as defined as PCV < 30%) was present in 25.3% (3.7%: moderate anaemia and 29.2%: mild anaemia). The geometric mean parasite density was not significantly different in the three communities: for the urban 641/*μ*L, for the periurban 930/*μ*L, and for the rural 592/*μ*L (*P* = 0.101). The rural community had the highest mean PCV of 34.1%; *P* = 0.001.

The malariometric indices for the AsyM carriers in the three communities are shown in Table 2. The rural community had the lowest rates of children with splenomegaly (6.3%), hepatomegaly (7.1%), and hepatosplenomegaly (4.7%) and these were statistically significant. The rural community also had the lowest proportion of children with anaemia (14.3%). The spleen rates, liver rates, and presence of hepatosplenomegaly and anaemia were similar in the urban and periurban communities.

There was no significant difference in anaemia between younger and older children in the three communities. There were however no significant differences in the presence of anaemia between older and younger children in the rural community. Splenomegaly (35.9% versus 21.1%), hepatomegaly (49.0% versus 23.7%), and hepatosplenomegaly (33.5% versus 12.9%) were significantly higher in the younger children in the total population. This was a consistent finding for hepatosplenomegaly in the urban (*P* < 0.0001) and periurban community (*P* = 0.031) but not in the rural community. Hepatomegaly was also associated with younger children in the urban and periurban population, *P* < 0.0001 and *P* = 0.003, respectively, but not in the rural population. Splenomegaly was associated with younger children only in the urban population (*P* = 0.041).

## 4. Discussion

Findings from our study show a high burden of malaria, with the parasite rates and liver and spleen rates being high especially in the urban and periurban communities. This is contrary to some previous studies which have shown that malaria transmission is generally lower in urban compared with rural areas [2, 3]. However, these studies only took into account urban areas with better quality housing and better access to healthcare facilities. However, in many densely populated urban slums where there is a high degree of poverty, malaria transmission remains high [5, 6]. Our study corroborates these findings with the urban area having the highest malaria burden with parasitaemia being positive and hepatosplenomegaly and anaemia being higher than in the periurban and rural areas.

Malaria transmission is influenced by many factors including the human reservoirs (asymptomatic carriers) for continued transmission of malaria. The prevalence of asymptomatic parasitaemia in children less than 48 months in areas with high malaria transmission varies in different populations. Bousema et al. [11] showed a high prevalence of asymptomatic asexual parasitaemia in about 74% of the population studied in western Kenya, while Gansane et al. [17] showed a prevalence of about 51% in a recent study carried out in Burkina Faso. Nkoghe et al. [18] showed 1.7–8.7% prevalence of asymptomatic carriers in a rural Gabonese population. We have shown a high incidence of asymptomatic carriers with more than half of the population studied being positive for malaria parasitaemia. The prevalence of asymptomatic carriers in this study is much lower than that from a study carried out by Udoh [5] in south-south Nigeria, who found a 71.4% prevalence, and from a previous study carried out in a rural community in Ibadan about 12 years ago, with a prevalence of 80% asymptomatic carriers [19].

Differences in malariometric indices display the unequal distribution of the risk and burden of malaria across different communities. The presence of a high number of asymptomatic carriers, especially children under the age of five, constitutes transmission reservoir and consequently influences the burden of malaria. Younger children had a higher prevalence of hepatomegaly, splenomegaly, and hepatosplenomegaly. These findings support already known facts that children of this age group are more susceptible to* P. falciparum* infection and the complicated forms [20, 21].

This study shows that malaria remains a major health problem among younger children. Malaria control should be intensified in Nigeria, especially in urban slums with crowded housing and poor environmental sanitation.

## Figures and Tables

**Table 1:** Characteristics of asymptomatic carriers in three communities in Ibadan.

Parameters	Three communities			*P*
	Urban	Periurban	Rural	
	*N* = 411	*N* = 119	*N* = 127	
Sex—males (%)	52.8	53.8	46.5	0.406
Age group % (under five)	62.8	52.9	34.6	0.000
Age median (range)	40.0 (107)	48.0 (112)	61.0 (116)	0.000
Parasite density (geometric mean)	641	930	592	0.101
Haematocrit (SD) (%)	32.2 (5.8)	31.5 (4.7)	34.1 (5.7)	0.001
White blood cell count (geometric mean)	5,865	7,990	8,156	0.000

**Table 2:** Malariometric indices of asymptomatic carriers in three communities.

Indices	Three communities			*χ* ^2^	*P*
	Urban	Periurban	Rural		
Mp positive	60.4	53.6	45.5	18.07	0.000
Hepatomegaly (present)	50.6	47.1	7.1	77.48	0.000
Splenomegaly (present)	38.0	36.1	6.3	46.49	0.000
Hepatosplenomegaly (present)	33.6	30.3	4.7	41.19	0.000
Anaemic %	29.0	24.6	14.3	10.98	0.004
